# Factors Involved in the Development of Diabetic Kidney Disease in Patients With Slowly Progressive Type 1 Diabetes Mellitus: A Retrospective Cohort Study

**DOI:** 10.7759/cureus.71055

**Published:** 2024-10-08

**Authors:** Hideyuki Okuma, Takahiro Tsutsumi, Masashi Ichijo, Tetsuro Kobayashi, Kyoichiro Tsuchiya

**Affiliations:** 1 Department of Diabetes and Endocrinology, Graduate School of Interdisciplinary Research, Faculty of Medicine, University of Yamanashi, Yamanashi, JPN; 2 Department of Endocrinology and Metabolism, Toranomon Hospital, Tokyo, JPN; 3 Diabetes and Endocrinology, University of Yamanashi Hospital, Yamanashi, JPN

**Keywords:** diabetic kidney disease (dkd), diabetic nephropathy (dn), dpp4 activity, high-sensitivity c-reactive protein (hscrp), slowly progressive type 1 insulin-dependent diabetes mellitus (spiddm), type i diabetes mellitus

## Abstract

Introduction

The duration of diabetes mellitus (DM), blood pro-inflammatory markers, and dipeptidyl peptidase 4 (DPP4) activity are known predictors of diabetic kidney disease (DKD) progression in acute-onset type 1 DM (AT1DM) and type 2 DM. However, predictors of DKD progression in slowly progressive type 1 insulin-dependent DM (SPIDDM) have been less frequently studied.

Patients and methods

This retrospective cohort study included 60 patients with SPIDDM (definite) (26 men/34 women). We utilized Cox proportional hazard analyses to determine whether characteristics and laboratory findings at the time of the SPIDDM diagnosis were associated with subsequent DKD progression. The urinary albumin-to-creatinine ratio (UACR) and estimated glomerular filtration rate (eGFR) at the last outpatient clinic visit served as indicators of renal function. Also, to compare blood markers in patients with SPIDDM, we included 21 patients diagnosed with AT1DM at the same department during the same period and 50 healthy adult volunteers.

Results

In patients with SPIDDM (definite), the multivariate Cox proportional hazard analysis revealed that the body mass index (BMI), high-sensitivity C-reactive protein (hs-CRP) levels at diagnosis, and the duration of DM prior to SPIDDM diagnosis were associated with the new onset of albuminuria and systolic blood pressure (SBP) at diagnosis, and the duration of DM prior to SPIDDM diagnosis was associated with a decline in eGFR to less than 60 ml/min/1.73 m². Additionally, serum hs-CRP levels were significantly higher in the SPIDDM (definite) group compared to both the AT1DM patients and healthy controls, suggesting a higher inflammatory state in patients with SPIDDM at the time of diagnosis. In patients with SPIDDM (definite) who were not treated with a DPP4 inhibitor, plasma DPP4 activity was associated with the new onset of albuminuria.

Conclusions

BMI, SBP, hs-CRP levels, DPP4 activity at SPIDDM diagnosis, and the duration of DM prior to SPIDDM diagnosis are associated with subsequent progression of DKD in SPIDDM. Also, we found that the patients with SPIDDM are often already being treated as DM for a long period of time at the time of diagnosis, which may be linked to their already high inflammatory status at the time of SPIDDM diagnosis and contribute to DKD progression. These findings underscore the importance of early diagnosis and management in preventing DKD progression in this population.

## Introduction

Diabetic nephropathy (DN) is one of the most severe complications of type 2 diabetes mellitus (T2DM) and acute-onset type 1 diabetes mellitus (AT1DM), with its prevalence on the rise [1]. Until recently, the traditional concept of DN was that urinary albumin excretion progressively increases over time as the glomerular filtration rate (GFR) declines, leading to end-stage renal disease (ESRD) [2]. However, recent trends show a reduction in cases of progressive albuminuria, while the number of patients experiencing a decline in GFR continues to increase [3]. The prevalence of early GFR decline before worsening albuminuria is notably rising. Factors such as aging, hypertension, and dyslipidemia, alongside diabetes management, influence the progression of diabetes-related kidney disease, also known as diabetic kidney disease (DKD). Given the increased incidence of cardiovascular events associated with deteriorating renal function [4] and the significant correlation between DKD and mortality in both T2DM and AT1DM [5,6], predicting the progression of DKD is a critical clinical challenge.

To date, the duration of diabetes mellitus (DM) and the degree of hypertension have been identified as predictors of DKD progression in T2DM [7]. For AT1DM, cumulative HbA1c levels during DM, initial HbA1c levels, and systolic blood pressure (SBP) at diagnosis have been recognized as predictors of DKD progression [8,9]. Furthermore, inflammatory cytokines have been reported to be involved in the pathogenesis of DN [10], and blood pro-inflammatory markers such as high-sensitivity C-reactive protein (hs-CRP), soluble receptor for advanced glycation end products (sRAGE), and advanced glycation end products (AGEs) have been reported to predict DKD progression in AT1DM and T2DM [11,12]. Additionally, dipeptidyl peptidase 4 (DPP4) activity is emerging as a potential predictor of DKD progression in these populations [13,14].

Among T1DM cases, slowly progressive type 1 insulin-dependent DM (SPIDDM) is characterized by the presence of islet-associated autoantibodies, such as anti-glutamic acid decarboxylase (anti-GAD) antibodies, and an absence of ketosis/ketoacidosis at the time of diagnosis [15,16]. In 2008, it was reported that the progression of diabetic nephropathy was less in latent autoimmune diabetes in adults, which is a concept similar to SPIDDM, compared to T2DM [17]; there have been no studies on the factors associated with DN and DKD progression in SPIDDM. Therefore, this retrospective cohort study aimed to determine whether characteristics and laboratory findings, including blood pro-inflammatory markers and DPP4 activity, at the time of SPIDDM diagnosis, were associated with subsequent DKD progression in patients who were finally diagnosed with SPIDDM (definite).

## Materials and methods

Patients

**Figure 1:**
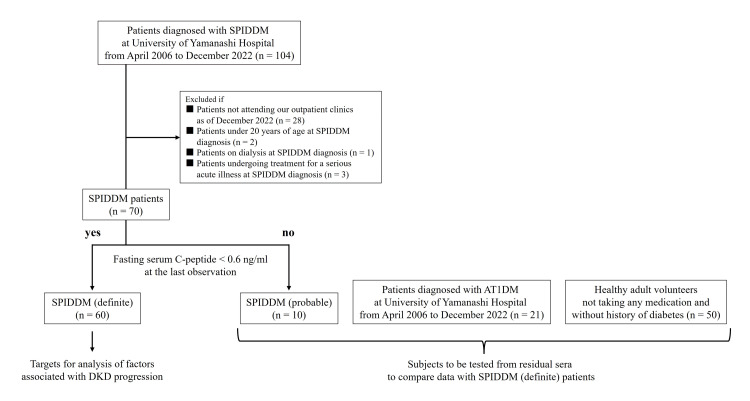
Flow chart of patient selection in this study. Specifically, after 34 patients were excluded from a total of 104 patients with SPIDDM according to the set exclusion criteria, the remaining 70 patients were classified as having SPIDDM (definite) or SPIDDM (probable) according to the SPIDDM diagnostic criteria of the Japan Diabetes Society. Among these 70 patients, 60 patients with SPIDDM (definite) were included in the analysis for factors associated with diabetic kidney disease progression. Further, 10 SPIDDM (probable) patients, 21 AT1DM patients, and 50 healthy adult volunteers were tested from residual serum to compare data with SPIDDM (definite) patients. SPIDDM, slowly progressive type 1 insulin-dependent diabetes mellitus; AT1DM, acute-onset type 1 diabetes mellitus

Of the 70 patients (34 men and 36 women) diagnosed with SPIDDM (probable) or SPIDDM (definite) at the Department of Diabetes and Endocrinology, University of Yamanashi Hospital, Yamanashi, Japan, between April 2006 and December 2022 and attending the outpatient clinic of the same department as of December 2022, 60 patients (26 men and 34 women) diagnosed with SPIDDM (definite) were included in the analysis of factors associated with DKD progression using residual serum (Figure 1). The remaining 10 patients, diagnosed with SPIDDM (probable), also had their residual serum analyzed for comparison with the SPIDDM (definite) group (Figure 1). SPIDDM diagnoses adhered to the latest guidelines from the Japan Diabetes Society (JDS), published in 2023 [16]. Patients aged < 20 years old, those on dialysis, or those being treated for serious acute illnesses at the time of the SPIDDM diagnosis were excluded from the study.

To compare blood pro-inflammatory markers and DPP4 activity, we included 21 patients diagnosed with AT1DM at the same department during the same period who were still attending the outpatient clinic as of December 2022. Additionally, 50 healthy adult volunteers (control group) not on any medication and with no history of diabetes, hypertension, dyslipidemia, or atherosclerotic diseases, who had participated in a previous study [18] and underwent medical check-ups at Isawa Hot Spring Hospital, Yamanashi, Japan, were also included. Similar to the SPIDDM patients, tests were conducted on residual serum and plasma samples from these individuals. Clinical autoimmune thyroid disease (AITD) was defined as either Graves’ disease treated with antithyroid agents or Hashimoto’s thyroiditis managed with hormone replacement therapy. This study received approval from the certified review boards of the University of Yamanashi and Isawa Hot Spring Hospital. Informed consent was obtained from all participants using an online opt-out option, allowing participants to decline participation by selecting this method.

Methods

**Figure 2:**
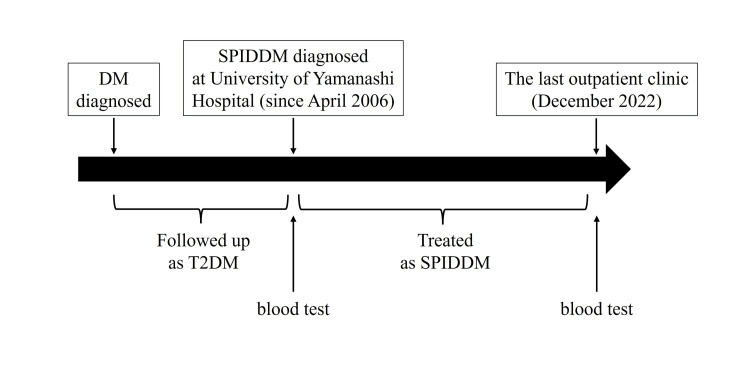
The timeline of this study. DM, diabetes mellitus; T2DM, type 2 diabetes mellitus; SPIDDM, slowly progressive type 1 insulin-dependent diabetes mellitus

The timeline of this study is depicted in Figure 2. To investigate factors associated with subsequent progression of DKD, data were extracted from the medical records at the time of SPIDDM diagnosis. The introduction of insulin therapy was determined by the attending physician based on the need to improve glycemic control or the presence of islet-associated autoantibodies. Anti-GAD antibodies were measured using an enzyme-linked immunosorbent assay (ELISA) provided by Cosmic Co., Tokyo, Japan, while anti-insulinoma-associated antigen-2 (anti-IA-2) antibodies were assessed using radioimmune assay kits from the same company. Endogenous insulin secretion capacity was evaluated using the C-peptide index (CPI), calculated as the fasting C-peptide (ng/ml) divided by fasting blood glucose (mg/dl) × 100. Additionally, residual blood samples from the patients were utilized to measure serum hs-CRP and plasma DPP4 activity. Serum hs-CRP levels were quantified using the immunoturbidimetric CRP-Latex assay according to the protocol from Kamiya Biomedical Company (Kamiya Biomedical Co., Seattle, Washington, United States).

Plasma DPP4 activity was determined based on the rate of cleavage of 7-amino-4-methylcoumarin (AMC) from the synthetic substrate H-glycyl-prolyl-AMC (H-Gly-Pro-AMC; Biovision, San Francisco, California, United States), and expressed as nmol of AMC cleaved per minute per ml (nmol/min/ml). Measurements of serum hs-CRP and plasma DPP4 activity were performed in duplicate, with any discrepancies outside of three standard deviations prompting reanalysis. For indicators of renal function, the urinary albumin-to-creatinine ratio (UACR) and estimated glomerular filtration rate (eGFR) values from the last outpatient clinic visit were used. Eligible SPIDDM patients were stratified into three groups based on UACR [19]: a normoalbuminuria group (UACR < 30 mg/g), a microalbuminuria group (30 mg/g ≤ UACR < 300 mg/g), and a macroalbuminuria group (UACR ≥ 300 mg/g). Similarly, patients were divided according to eGFR [19] into two categories: eGFR ≥ 60 ml/min/1.73 m^2^ and eGFR < 60 ml/min/1.73 m^2^. Cox proportional hazard analysis excluded patients who had a UACR ≥ 30 mg/g (n = 1) and an eGFR < 60 ml/min/1.73 m^2^ (n = 3) at the time of SPIDDM diagnosis.

Statistical analysis

Continuous variables with a normal distribution were analyzed using the t-test and analysis of variance (ANOVA), while those not normally distributed were evaluated using the Mann-Whitney U test and the Kruskal-Wallis test. Nominal variables were analyzed using Fisher’s exact test. Pearson’s correlation coefficient was employed for correlation analyses. Cox proportional hazard analyses were conducted to identify factors associated with DKD progression in SPIDDM patients. Kaplan-Meier survival curves were generated, and differences between groups were assessed using the log-rank test. All statistical analyses were performed using Prism 7 software (GraphPad Software Inc., San Diego, California, United States), with statistical significance set at P < 0.05.

## Results

Comparison of clinical characteristics by classification based on UACR at the time of the last outpatient clinic visit

Initially, the 60 patients with SPIDDM (definite), who were eligible for analysis of predictors of DKD progression, were divided into three groups based on their UACR at the last outpatient clinic visit: normoalbuminuria, microalbuminuria, and macroalbuminuria (Table 1). No significant differences in sex or age were observed between the groups. Body mass index (BMI) and hs-CRP levels at the time of SPIDDM diagnosis were higher in groups with elevated UACR, while the CPI at diagnosis was lower in these groups. Additionally, the duration of DM prior to SPIDDM diagnosis was significantly longer in the groups with higher UACR, though the duration of SPIDDM itself did not differ significantly. There were also no significant differences in the medications prescribed among these groups (Table 2).

**Table 1:** Clinical characteristics of SPIDDM (definite) patients classified by UACR at the time of last outpatient clinic visit. Data are expressed as the mean ± standard deviation, the median (range), or number (%), and are compared using the one-way analysis of variance (ANOVA) test, the Kruskal-Wallis test, or the Fisher’s exact test. Hyperglycemic symptoms include thirst, polyuria, and body weight loss. Items without a time statement are at SPIDDM diagnosis. SPIDDM, slowly progressive type 1 insulin-dependent diabetes mellitus; N/A, not available; BMI, body mass index; SBP, systolic blood pressure; DM, diabetes mellitus; AITD, autoimmune thyroid disease; anti-GAD, anti-glutamic acid decarboxylase; anti-IA-2, anti-insulinoma-associated antigen-2; HbA1c, hemoglobin A1c; UACR, urine albumin-to-creatinine ratio; eGFR, estimated glomerular filtration rate; HDL-C, high-density lipoprotein cholesterol; LDL-C, low-density lipoprotein cholesterol; TG, triglycerides; UA, uric acid; hs-CRP, high-sensitivity C-reactive protein

	Overall (n=60)			
	Normoalbuminuria	Microalbuminuria	Macroalbuminuria	P
Subjects (n)	30	17	13	N/A
Females (n, %)	20 (67)	9 (53)	5 (39)	0.195
Age at the last outpatient clinic (years)	56.3±14.8	59.5±17.4	59.5±15.0	0.726
Age at SPIDDM diagnosis (years)	46.3±14.8	49.9±17.6	49.4±14.8	0.7
Smoking (n, %)	12 (40)	5 (29)	3 (23)	0.594
BMI (kg/m^2^)	21.9±2.64	26.8±2.32	27.5±3.07	<0.001
SBP (mmHg)	122 (104-178)	120 (101-163)	143 (104-158)	0.886
Duration of DM prior to SPIDDM diagnosis (years)	3 (0-7)	6 (0-11)	8 (1-13)	<0.001
Duration of SPIDDM (years)	8.5 (1-24)	9 (1-19)	8 (3-19)	0.965
Time from DM diagnosis to introduction of insulin therapy (years)	3 (0-17)	4 (2-8)	2 (1-14)	0.117
Hyperglycemic symptoms (n, %)	16 (53)	9 (53)	11 (85)	0.136
Family history of DM (n, %)	19 (63)	11 (65)	8 (62)	1
Co-occurrence of clinical AITD (n, %)	6 (20)	9 (53)	3 (23)	0.055
Chronic thyroiditis	6 (20)	9 (53)	3 (23)	0.055
Graves’ disease	0 (0)	0 (0)	0 (0)	1
Anti-GAD antibody titer (U/ml)	32.5 (5-1432)	25.0 (5-352)	46.0 (8-890)	0.597
Anti-IA-2 Ab positive (n, %)	7 (23)	5 (29)	5 (39)	0.606
C-peptide index	1.325 (0.74-2.9)	0.99 (0.61-1.55)	0.96 (0.42-1.62)	<0.001
HbA1c (%)	9.3 (6.5-15.9)	10.2 (6.9-12.9)	9.4 (7.2-14.1)	0.827
UACR at SPIDDM diagnosis (mg/g)	5.15 (1.5-26.4)	4.3 (1.2-19.4)	3.4 (1-211)	0.293
eGFR at SPIDDM diagnosis (ml/min/1.73m^2^)	79.6±11.5	77.2±7.30	75.1±18.8	0.536
HDL-C (mg/dl)	52 (34-79)	62 (36-79)	44 (33-79)	0.139
LDL-C (mg/dl)	119 (78-169)	116 (84-199)	120 (70-174)	0.84
TG (mg/dl)	114 (44-347)	137 (80-340)	134 (84-210)	0.1
UA (mg/dl)	6.01±1.41	5.83±1.64	5.65±1.15	0.736
hs-CRP (mg/l)	3.25 (1.7-7.0)	4.90 (2.7-12.5)	7.50 (3.1-13.8)	<0.001

**Table 2:** Characteristics of the prescribed drugs for SPIDDM (definite) patients classified by UACR at the time of last outpatient clinic. Data are expressed as number (%) and are compared using Fisher's exact test. GLP-1, glucagon-like peptide-1; DPP4, dipeptidyl peptidase-4; SGLT2, sodium glucose co-transporter 2; ACE, angiotensin-converting enzyme; ARB, angiotensin receptor blockers; SPIDDM, slowly progressive type 1 insulin-dependent diabetes mellitus; DM, diabetes mellitus

	Overall (n=60)			
	Normoalbuminuria	Microalbuminuria	Macroalbuminuria	P
Treatment for DM before SPIDDM diagnosis (n, %)				
Drug-free	4 (13)	1 (6)	0 (0)	0.488
Insulin	10 (33)	7 (41)	2 (15)	0.341
GLP-1 analog	0 (0)	0 (0)	0 (0)	1
Oral hypoglycemic agents				
Sulfonylurea	7 (23)	7 (41)	4 (31)	0.469
Glinide	7 (23)	3 (18)	4 (31)	0.673
DPP4 inhibitor	10 (33)	2 (12)	5 (38)	0.2
Biguanide	8 (27)	4 (24)	6 (46)	0.352
Thiazolidine	4 (13)	2 (12)	2 (15)	1
α-glucosidase inhibitor	4 (13)	3 (18)	2 (15)	0.898
SGLT2 inhibitor	1 (3)	3 (18)	1 (8)	0.167
Treatment for hypertension before SPIDDM diagnosis (n, %)				
ACE inhibitor /ARB	5 (17)	5 (29)	3 (23)	0.601
Anti-hypertensive agent (except for ACE inhibitor and ARB)	4 (13)	4 (24)	5 (38)	0.17
Treatment for dyslipidemia before SPIDDM diagnosis (n, %)				
Statin	8 (27)	5 (29)	3 (23)	1
Fibrate	5 (17)	3 (18)	1 (8)	0.799
Treatment for DM at the time of last outpatient clinic (n, %)				
Drug-free	0 (0)	0 (0)	0 (0)	1
Insulin	30 (100)	17 (100)	13 (100)	1
GLP-1 analog	7 (23)	3 (18)	4 (31)	0.673
Oral hypoglycemic agents				
Sulfonylurea	0 (0)	0 (0)	0 (0)	1
Glinide	0 (0)	0 (0)	0 (0)	1
DPP4 inhibitor	6 (20)	1 (6)	2 (15)	0.46
Biguanide	13 (43)	5 (29)	5 (38)	0.693
Thiazolidine	6 (20)	3 (18)	3 (23)	1
α-glucosidase inhibitor	2 (7)	2 (12)	2 (15)	0.628
SGLT2 inhibitor	7 (23)	6 (35)	4 (31)	0.699
Treatment for hypertension at the time of last outpatient clinic (n, %)				
ACE inhibitor / ARB	10 (33)	9 (53)	9 (69)	0.0896
Anti-hypertensive agent (except for ACE inhibitor and ARB)	8 (27)	2 (12)	5 (38)	0.245
Treatment for dyslipidemia at the time of last outpatient clinic (n, %)				
Statin	13 (43)	8 (47)	5 (38)	0.943
Fibrate	7 (23)	5 (29)	4 (31)	0.799

Comparison of clinical characteristics by classification based on eGFR at the time of the last outpatient clinic visit

Subsequently, the same cohort of 60 SPIDDM patients was categorized into two groups based on their eGFR at the last outpatient clinic visit (Table 3). Similar to the UACR classification, no significant differences in sex or age were found between these groups. However, SBP and hs-CRP levels at the time of SPIDDM diagnosis were significantly higher in the group with eGFR less than 60 ml/minute/1.73 m^2^. The duration of DM prior to SPIDDM diagnosis was also significantly longer in this group, but, as with the UACR comparison, the duration of SPIDDM itself showed no significant difference. No significant differences in prescribed medications were noted between the groups (Table 4).

**Table 3:** Clinical characteristics of SPIDDM (definite) patients classified by eGFR at the time of last outpatient clinic visit. Data are expressed as the mean ± standard deviation, the median (range), or number (%), and are compared using the student’s t-test, the Mann-Whitney U test, or Fisher’s exact test. Items without a time statement are at SPIDDM diagnosis. SPIDDM, slowly progressive type 1 insulin-dependent diabetes mellitus; BMI, body mass index; SBP, systolic blood pressure; DM, diabetes mellitus; AITD, autoimmune thyroid disease; anti-GAD, anti-glutamic acid decarboxylase; anti-IA-2, anti-insulinoma-associated antigen-2; HbA1c, hemoglobin A1c; UACR, urine albumin-to-creatinine ratio; eGFR, estimated glomerular filtration rate; HDL-C, high-density lipoprotein cholesterol; LDL-C, low-density lipoprotein cholesterol; TG, triglycerides; UA, uric acid; hs-CRP, high-sensitivity C-reactive protein

	Overall (n=60)		
	eGFR ≥ 60	eGFR < 60	P
Subjects (n)	31	29	N/A
Females (n, %)	20 (59)	14 (41)	0.297
Age at the last outpatient clinic (years)	56.5±12.9	59.3±17.9	0.483
Age at SPIDDM diagnosis (years)	45.7±13.1	50.4±17.5	0.239
Smoking (n, %)	9 (29)	11 (38)	0.586
BMI (kg/m^2^)	24.0±3.3	25.1±4.1	0.27
SBP (mmHg)	117 (101-142)	154 (106-178)	<0.001
Duration of DM prior to SPIDDM diagnosis (years)	3 (0-9)	7 (0-13)	<0.001
Duration of SPIDDM (years)	9 (3-24)	8 (1-19)	0.221
Time from DM diagnosis to introduction of insulin therapy (years)	4 (0-11)	2 (0-17)	0.063
Hyperglycemic symptoms (n, %)	18 (58.1)	18 (62.1)	0.797
Family history of DM (n, %)	18 (58.1)	20 (69)	0.431
Co-occurrence of clinical AITD (n, %)	9 (29)	9 (31)	1
Chronic thyroiditis	9 (29)	9 (31)	1
Graves’ disease	0 (0)	0 (0)	1
Anti-GAD antibody titer (U/ml)	35 (5-1432)	24 (5-890)	0.108
Anti-IA-2 Ab positive (n, %)	11 (36)	6 (21)	0.258
C-peptide index	1.23 (0.61-2.5)	1.07 (0.42-2.9)	0.506
HbA1c (%)	9.3 (6.5-12.9)	9.4 (6.7-15.9)	0.888
UACR at SPIDDM diagnosis (mg/g)	4.5 (1.0-23.2)	5.1 (1.1-211)	0.75
eGFR at SPIDDM diagnosis (ml/min/1.73m^2^)	80.5±10.9	75.2±13.5	0.098
HDL-C (mg/dl)	52 (33-79)	46 (33-79)	0.203
LDL-C (mg/dl)	117 (78-199)	121 (70-199)	0.344
TG (mg/dl)	115 (44-347)	131 (58-335)	0.336
UA (mg/dl)	6.13±1.52	5.61±1.25	0.149
hs-CRP (mg/l)	3.9 (1.7-12.5)	5.0 (2.2-13.8)	0.021

**Table 4:** Characteristics of the prescribed drugs for SPIDDM (definite) patients classified by eGFR at the time of last outpatient clinic. Data are expressed as number (%) and are compared using Fisher's exact test. GLP-1, glucagon-like peptide-1; DPP4, dipeptidyl peptidase-4; SGLT2, sodium glucose co-transporter 2; ACE, angiotensin-converting enzyme; ARB, angiotensin receptor blockers; SPIDDM, slowly progressive type 1 insulin-dependent diabetes mellitus; DM, diabetes mellitus

	Overall (n=60)		
	eGFR ≥ 60	eGFR < 60	P
Treatment for DM (before SPIDDM diagnosis) (n, %)			
Drug-free	3 (10)	2 (7)	1
Insulin	6 (19)	13 (45)	0.0517
GLP-1 analog	0 (0)	0 (0)	1
Oral hypoglycemic agents			
Sulfonylurea	9 (29)	9 (31)	1
Glinide	7 (23)	7 (24)	1
DPP4 inhibitor	7 (23)	10 (35)	0.394
Biguanide	8 (26)	10 (35)	0.576
Thiazolidine	5 (16)	3 (10)	0.708
α-glucosidase inhibitor	3 (10)	6 (21)	0.292
SGLT2 inhibitor	2 (7)	3 (10)	0.666
Treatment for hypertension (before SPIDDM diagnosis) (n, %)			
ACE inhibitor / ARB	6 (19)	7 (24)	0.758
Anti-hypertensive agent (except for ACE inhibitor and ARB)	5 (16)	8 (28)	0.355
Treatment for dyslipidemia (before SPIDDM diagnosis) (n, %)			
Statin	7 (23)	9 (31)	0.563
Fibrate	7 (23)	2 (7)	0.148
Treatment for DM at the time of last outpatient clinic (n, %)			
Drug-free	0 (0)	0 (0)	1
Insulin	31 (100)	29 (100)	1
GLP-1 analog	6 (19)	8 (28)	0.547
Oral hypoglycemic agents			
Sulfonylurea	0 (0)	0 (0)	1
Glinide	0 (0)	0 (0)	1
DPP4 inhibitor	5 (16)	4 (14)	1
Biguanide	13 (42)	10 (34)	0.603
Thiazolidine	8 (26)	4 (14)	0.337
α-glucosidase inhibitor	2 (7)	4 (14)	0.417
SGLT2 inhibitor	7 (23)	10 (34)	0.394
Treatment for hypertension at the time of last outpatient clinic (n, %)			
ACE inhibitor / ARB	13 (42)	15 (52)	0.605
Anti-hypertensive agent	6 (19)	9 (31)	0.376
(except for ACE inhibitor and ARB)			
Treatment for dyslipidemia at the time of last outpatient clinic (n, %)			
Statin	12 (39)	14 (48)	0.603
Fibrate	10 (32)	6 (21)	0.387

Survival analysis of albuminuria and reduced eGFR in SPIDDM (definite) patients

We conducted survival analyses during the follow-up of SPIDDM to explore factors linked with the new onset of DKD in patients. Renal survival was defined as the absence of albuminuria or a decline in eGFR to less than 60 ml/minute/1.73 m² during the follow-up period as SPIDDM. Initially, we undertook a univariate Cox proportional hazard analysis, considering the new appearance of albuminuria or a decline in eGFR to less than 60 ml/minute/1.73 m² as the event (Table 5). In this analysis, BMI, CPI, hs-CRP levels at SPIDDM diagnosis, and the duration of DM prior to SPIDDM diagnosis exhibited significant hazard ratios for the new onset of albuminuria. Similarly, SBP at diagnosis and the duration of DM prior to SPIDDM diagnosis showed significant hazard ratios for a new decline in eGFR.

**Table 5:** Univariate Cox proportional hazard models for progression to DKD in SPIDDM (definite) patients. Items without a time statement are at SPIDDM diagnosis. HR, hazard ratio; CI, confidence interval; SPIDDM, slowly progressive type 1 insulin-dependent diabetes mellitus; BMI, body mass index; SBP, systolic blood pressure; DM, diabetes mellitus; AITD, autoimmune thyroid disease; anti-GAD, anti-glutamic acid decarboxylase; anti-IA-2, anti-insulinoma-associated antigen-2; HbA1c, hemoglobin A1c; UACR, urine albumin-to-creatinine ratio; eGFR, estimated glomerular filtration rate; HDL-C, high-density lipoprotein cholesterol; LDL-C, low-density lipoprotein cholesterol; TG, triglycerides; UA, uric acid; hs-CRP, high-sensitivity C-reactive protein; DKD, diabetic kidney disease

Event: New appearance of albuminuria			
Covariates	HR	95% CI	P
Age (years)	1.016	0.991-1.041	0.207
Sex (0: Males, 1: Females)	0.628	0.301-1.307	0.213
Smoking (0: no, 1: yes)	0.764	0.338-1.729	0.519
BMI (kg/m^2^)	1.331	1.194-1.485	<0.001
SBP (mmHg)	1.001	0.984-1.019	0.880
Duration of DM prior to SPIDDM diagnosis(years)	1.419	1.245-1.618	<0.001
Duration of SPIDDM (years)	0.948	0.879-1.022	0.163
Time from DM diagnosis to introduction of insulin therapy (years)	1.028	0.932-1.134	0.586
C-peptide index	0.118	0.035-0.400	<0.001
HbA1c (%)	1.018	0.857-1.210	0.835
UACR at SPIDDM diagnosis (mg/g)	0.989	0.935-1.047	0.711
eGFR at SPIDDM diagnosis (ml/min/1.73m^2^)	0.978	0.948-1.008	0.151
HDL-C (mg/dl)	0.998	0.972-1.025	0.863
LDL-C (mg/dl)	1.005	0.993-1.017	0.412
TG (mg/dl)	1.002	0.998-1.007	0.314
UA (mg/dl)	0.854	0.656-1.111	0.239
hs-CRP (mg/l)	1.322	1.178-1.483	<0.001
Event: New decline in eGFR to less than 60 ml/min/1.73 m^2^			
Covariates	HR	95% CI	P
Age (years)	1.014	0.987-1.041	0.326
Sex (0: Males, 1: Females)	0.691	0.320-1.491	0.347
Smoking (0: no, 1: yes)	1.253	0.569-2.763	0.575
BMI (kg/m^2^)	1.043	0.931-1.169	0.466
SBP (mmHg)	1.064	1.039-1.088	<0.001
Duration of DM prior to SPIDDM diagnosis(years)	1.228	1.088-1.385	<0.001
Duration of SPIDDM (years)	0.934	0.861-1.012	0.096
Time from DM diagnosis to introduction of insulin therapy (years)	0.951	0.826-1.095	0.485
C-peptide index	0.942	0.419-2.119	0.886
HbA1c (%)	0.994	0.816-1.211	0.952
UACR at SPIDDM diagnosis (mg/g)	1.009	0.999-1.019	0.093
eGFR at SPIDDM diagnosis (ml/min/1.73m^2^)	0.992	0.957-1.028	0.664
HDL-C (mg/dl)	0.985	0.957-1.015	0.319
LDL-C (mg/dl)	1.004	0.992-1.016	0.517
TG (mg/dl)	1.001	0.996-1.007	0.702
UA (mg/dl)	0.896	0.693-1.158	0.400
hs-CRP (mg/l)	1.081	0.952-1.226	0.229

**Figure 3:**
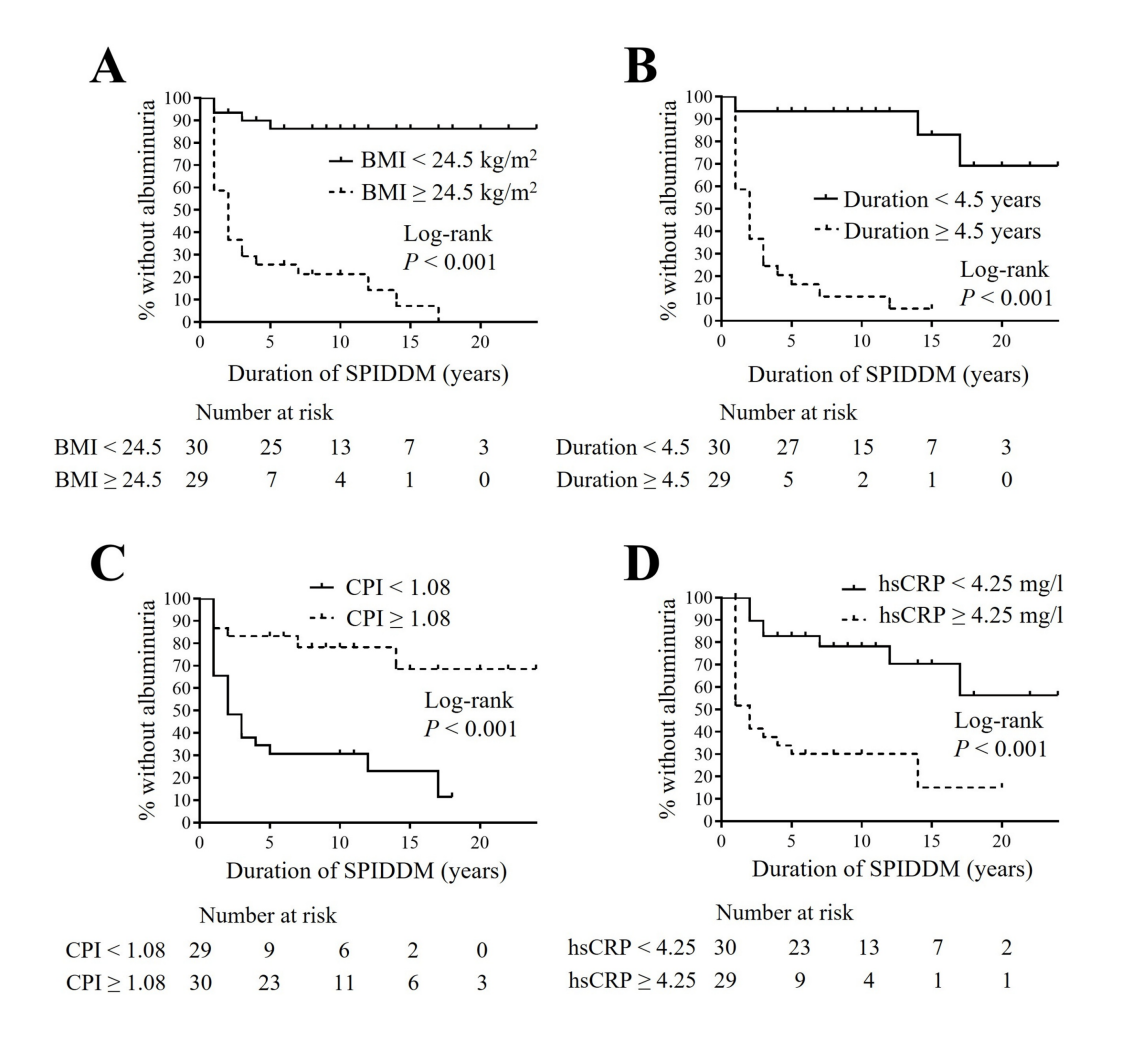
Kaplan-Meier survival curves for the new appearance of albuminuria during follow-up for SPIDDM according to BMI with cut-off ≤ 24.5 kg/m2 (A), duration of DM prior to SPIDDM diagnosis with cut-off ≤ 4.5 years (B), CPI with cut-off ≤ 1.08 (C), and hs-CRP with cut-off ≤ 4.25 mg/l (D). Items without a time statement are at SPIDDM diagnosis. BMI, body mass index; duration, duration of DM prior to SPIDDM diagnosis; CPI, C-peptide index; hs-CRP, high-sensitivity C-reactive protein; SPIDDM, slowly progressive type 1 insulin-dependent diabetes mellitus; DM, diabetes mellitus

**Figure 4:**
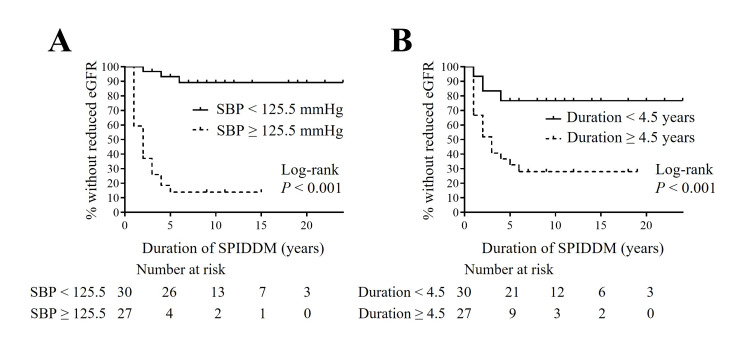
Kaplan-Meier survival curves for the new decline in eGFR to less than 60 ml/minute/1.73 m2 during follow-up for SPIDDM according to SBP with cut-off ≤ 125.5 mmHg (A) and duration of DM prior to SPIDDM diagnosis with cut-off ≤ 4.5 years (B). Items without a time statement are at SPIDDM diagnosis. eGFR, estimated glomerular filtration rate; SBP, systolic blood pressure; duration, duration of DM prior to SPIDDM diagnosis; SPIDDM, slowly progressive type 1 insulin-dependent diabetes mellitus; DM, diabetes mellitus

Subsequently, to illustrate the long-term impact of these factors on DKD progression, we employed the Kaplan-Meier method. Patients were divided into two groups based on the median or mean values of each parameter to optimize the power for statistical analysis. The group with higher BMI, a longer duration of DM prior to SPIDDM diagnosis, lower CPI, and higher hs-CRP levels developed microalbuminuria significantly earlier than those without these factors (Figure 3). Similarly, the group with higher SBP and a longer duration of DM prior to SPIDDM diagnosis experienced a significant earlier reduction in eGFR (Figure 4). Finally, we performed a multivariate Cox proportional hazard analysis (Tables 6, 7). The covariates included were those that showed significant hazard ratios in the univariate analysis (Table 5). After adjusting for previously reported risk factors for DKD, such as age, gender, smoking habits, and dyslipidemia, all covariates except CPI remained significant (Tables 6, 7) [7]. Additionally, in the model with these covariates entered simultaneously, BMI, hs-CRP levels at SPIDDM diagnosis, and duration of DM prior to SPIDDM diagnosis showed significant hazard ratios for the new onset of albuminuria (Table 6), while SBP at SPIDDM diagnosis and duration of DM prior to SPIDDM diagnosis showed significant hazard ratios for a new decline in eGFR (Table 7).

**Table 6:** Multivariate Cox proportional hazard models for the new appearance of albuminuria in SPIDDM (definite) patients. *All covariates, including BMI, duration of DM until SPIDDM diagnosis, CPI, and hs-CRP, are entered simultaneously into the model. Items without a time statement are at SPIDDM diagnosis. SPIDDM, slowly progressive type 1 insulin-dependent diabetes mellitus; DM, diabetes mellitus; BMI, body mass index; SBP, systolic blood pressure; HbA1c, hemoglobin A1c; UACR, urine albumin-to-creatinine ratio; eGFR, estimated glomerular filtration rate; HDL-C, high-density lipoprotein cholesterol; LDL-C, low-density lipoprotein cholesterol; TG, triglycerides; UA, uric acid; hs-CRP, high-sensitivity C-reactive protein; HR, hazard ratio; CI, confidence interval

Event: New appearance of albuminuria												
	Covariates											
	BMI (kg/m^2^)			Duration of DM prior to SPIDDM diagnosis (years)			C-peptide index			hs-CRP (mg/l)		
	HR	95% CI	P	HR	95% CI	P	HR	95% CI	P	HR	95% CI	P
Age-adjusted HR	1.390	1.224-1.579	< 0.001	1.444	1.261-1.654	< 0.001	0.118	0.037-0.376	< 0.001	1.325	1.178-1.491	< 0.001
Sex-adjusted HR	1.327	1.188-1.482	< 0.001	1.433	1.242-1.652	< 0.001	0.118	0.033-0.424	0.001	1.319	1.166-1.491	< 0.001
Smoking-adjusted HR	1.335	1.190-1.499	< 0.001	1.424	1.245-1.629	< 0.001	0.119	0.034-0.412	< 0.001	1.320	1.175-1.483	< 0.001
BMI-adjusted HR	N/A	N/A	N/A	1.282	1.101-1.494	0.001	0.318	0.087-1.165	0.084	1.212	1.071-1.371	0.002
SBP-adjusted HR	1.343	1.200-1.503	< 0.001	1.419	1.244-1.618	< 0.001	0.119	0.035-0.401	< 0.001	1.335	1.186-1.504	< 0.001
Duration of DM prior to SPIDDM diagnosis-adjusted HR	1.188	1.054-1.339	0.005	N/A	N/A	N/A	0.345	0.095-1.247	0.105	1.158	1.003-1.338	0.046
C-peptide index-adjusted HR	1.263	1.121-1.422	< 0.001	1.349	1.170-1.556	< 0.001	N/A	N/A	N/A	1.239	1.103-1.391	< 0.001
HbA1c-adjusted HR	1.334	1.195-1.490	< 0.001	1.420	1.246-1.620	< 0.001	0.105	0.028-0.386	< 0.001	1.322	1.178-1.483	< 0.001
UACR at SPIDDM diagnosis-adjusted HR	1.337	1.195-1.496	< 0.001	1.418	1.244-1.616	< 0.001	0.106	0.029-0.393	< 0.001	1.319	1.177-1.478	< 0.001
eGFR at SPIDDM diagnosis-adjusted HR	1.320	1.185-1.471	< 0.001	1.459	1.252-1.700	< 0.001	0.103	0.028-0.375	< 0.001	1.329	1.182-1.495	< 0.001
HDL-C-adjusted HR	1.336	1.196-1.492	< 0.001	1.420	1.246-1.618	< 0.001	0.110	0.030-0.404	< 0.001	1.328	1.180-1.496	< 0.001
LDL-C-adjusted HR	1.399	1.233-1.587	< 0.001	1.421	1.246-1.622	< 0.001	0.112	0.032-0.396	< 0.001	1.322	1.177-1.484	< 0.001
TG-adjusted HR	1.341	1.200-1.499	< 0.001	1.415	1.241-1.613	< 0.001	0.104	0.028-0.381	< 0.001	1.320	1.174-1.484	< 0.001
hs-CRP-adjusted HR	1.272	1.134-1.426	< 0.001	1.304	1.122-1.516	< 0.001	0.172	0.045-0.657	0.010	N/A	N/A	N/A
Multivariate-adjusted HR*	1.162	1.030-1.312	0.015	1.180	1.008-1.380	0.039	0.491	0.127-1.903	0.304	1.144	1.004-1.304	0.044

**Table 7:** Multivariate Cox proportional hazard models for the new decline in eGFR to less than 60 ml/minute/1.73 m2 in SPIDDM (definite) patients. *All covariates, including SBP and duration DM until SPIDDM diagnosis, are entered simultaneously into the model. Items without a time statement are at SPIDDM diagnosis. SPIDDM, slowly progressive type 1 insulin-dependent diabetes mellitus; DM, diabetes mellitus; SBP, systolic blood pressure; BMI, body mass index; HbA1c, hemoglobin A1c; UACR, urine albumin-to-creatinine ratio; eGFR, estimated glomerular filtration rate; HDL-C, high-density lipoprotein cholesterol; LDL-C, low-density lipoprotein cholesterol; TG, triglycerides; UA, uric acid; hs-CRP, high-sensitivity C-reactive protein; HR, hazard ratio; CI, confidence interval

Event: New decline in eGFR to less than 60 ml/min/1.73 m^2^						
	Covariates					
	SBP (mmHg)			Duration of DM prior to SPIDDM diagnosis (years)		
	HR	95% CI	P	HR	95% CI	P
Age-adjusted HR	1.065	1.040-1.091	< 0.001	1.259	1.108-1.431	< 0.001
Sex-adjusted HR	1.063	1.039-1.088	< 0.001	1.220	1.078-1.380	0.002
Smoking-adjusted HR	1.065	1.040-1.091	< 0.001	1.244	1.101-1.405	< 0.001
BMI-adjusted HR	1.064	1.040-1.089	< 0.001	1.265	1.107-1.446	< 0.001
HbA1c-adjusted HR	1.064	1.039-1.088	< 0.001	1.229	1.088-1.387	< 0.001
C-peptide index-adjusted HR	1.067	1.041-1.094	< 0.001	1.256	1.104-1.430	< 0.001
UACR at SPIDDM diagnosis-adjusted HR	1.064	1.040-1.089	< 0.001	1.219	1.074-1.382	0.002
eGFR at SPIDDM diagnosis-adjusted HR	1.065	1.040-1.090	< 0.001	1.234	1.090-1.398	< 0.001
HDL-C-adjusted HR	1.063	1.038-1.087	< 0.001	1.222	1.083-1.380	0.001
LDL-C-adjusted HR	1.064	1.040-1.089	< 0.001	1.230	1.087-1.391	0.001
TG-adjusted HR	1.064	1.040-1.089	< 0.001	1.228	1.088-1.386	< 0.001
hs-CRP-adjusted HR	1.067	1.042-1.092	< 0.001	1.233	1.075-1.414	0.003
Multivariate-adjusted HR*	1.064	1.039-1.089	< 0.001	1.274	1.096-1.482	0.002

Comparison of hs-CRP and other clinical characteristics at diagnosis by classification based on disease type

**Figure 5:**
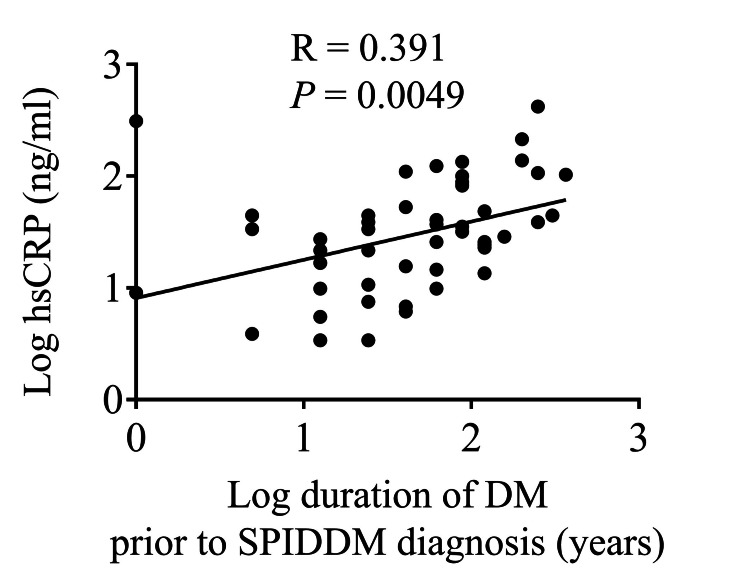
Correlation between the duration of DM prior to SPIDDM diagnosis and hs-CRP level. Pearson’s correlation coefficient analysis was performed to analyze the correlation between the duration of DM prior to SPIDDM diagnosis and hs-CRP levels. For the correlation analysis, these variables were log-transformed. Items without a time statement are at SPIDDM diagnosis. R, R correlation coefficient; hs-CRP, high-sensitivity C-reactive protein; SPIDDM; slowly progressive type 1 insulin-dependent diabetes mellitus; DM; diabetes mellitus

The data presented earlier indicate that inflammation-related markers, such as hs-CRP, at the time of SPIDDM diagnosis may be linked to subsequent progression of DKD. Given previous studies highlighting a correlation between the duration of DM and levels of inflammation-related markers [20], we conducted a correlation analysis between hs-CRP levels at SPIDDM diagnosis and the duration of DM leading up to SPIDDM diagnosis. The results demonstrated a significant positive correlation between hs-CRP levels and the duration of DM prior to SPIDDM diagnosis (Figure 5). Further, we compared hs-CRP levels among patients with SPIDDM (definite), SPIDDM (probable), AT1DM patients, and healthy controls (Table 8). Samples from SPIDDM and AT1DM patients were collected at the time of diagnosis for each respective disease type. There were no significant differences in hs-CRP levels between SPIDDM (definite) and SPIDDM (probable) patients at the time of diagnosis. However, hs-CRP levels were significantly higher in the SPIDDM (definite) group compared to both the AT1DM patients and healthy controls, suggesting a higher inflammatory state in patients with SPIDDM at the time of diagnosis.

**Table 8:** Comparison of hs-CRP and other clinical characteristics at diagnosis by classification based on disease type. Data are expressed as the mean ± standard deviation, the median (range), or number (%), and are compared using the student’s t-test, the Mann-Whitney U test, or Fisher's exact test. *P < 0.05 vs. SPIDDM (propable). ##P < 0.01, ###P < 0.001 vs. AT1DM. ††P < 0.01, †††P < 0.001 vs. healthy controls. Items without a time statement are at SPIDDM diagnosis. SPIDDM, slowly progressive type 1 insulin-dependent diabetes mellitus; BMI, body mass index; SBP, systolic blood pressure; HbA1c, hemoglobin A1c; UACR, urine albumin-to-creatinine ratio; eGFR, estimated glomerular filtration rate; HDL-C, high-density lipoprotein cholesterol; LDL-C, low-density lipoprotein cholesterol; TG, triglycerides; UA, uric acid; hs-CRP, high-sensitivity C-reactive protein; AT1DM, acute-onset type 1 diabetes mellitus; N/A, not available

	SPIDDM (definite)	SPIDDM (probable)	AT1DM	Healthy controls
Subjects (n)	60	10	21	50
Females (n, %)	34 (57) ^*^	2 (20)	10 (48)	22 (44)
Age at the last outpatient clinic (years)	57.9±15.4 ^###^	60.4±9.34	36.4±13.1	N/A
Age at the time of collecting serum (years)	48.0±15.4 ^## †††^	50.4±9.40	35.4±11.4	61.8±8.49
BMI (kg/m^2^)	24.5±3.72 ^* ### ††^	27.0±2.36	21.1±2.77	22.5±2.89
SBP (mmHg)	125.5 (101-178) ^## †††^	131.5 (120-154)	113 (100-132)	117 (94-138)
Duration of SPIDDM or AT1DM (years)	8.5 (1-24)	10 (5-15)	9 (2-34)	N/A
Duration of DM prior to the time of collecting serum (years)	4.5 (0-13) ^###^	5 (0-12)	0 (0-8)	N/A
C-peptide index	1.08 (0.42-2.9) ^###^	1.37 (0.84-2.17)	0.20 (0.07-0.5)	N/A
HbA1c (%)	9.3 (6.5-15.9) ^## †††^	9.2 (7.5-13.2)	11.5 (7.1-16.6)	5.6 (5.0-6.3)
UACR at the time of collecting serum (mg/g)	4.65 (1.0-211) ^*^	11.95 (2.2-32.9)	7.2 (1.1-69)	N/A
eGFR at the time of collecting serum (ml/min/1.73m^2^)	78.0±12.4 ^### ††^	75.3±6.68	103±32.5	71.4±9.66
UACR at the last outpatient clinic (mg/g)	29.8 (3.0-3000) ^###^	16.75 (4.1-81.1)	6.5 (1.9-1674)	N/A
eGFR at the last outpatient clinic (ml/min/1.73m^2^)	59.2±22.0 ^###^	64.2±10.8	81.9±21.4	N/A
hs-CRP (mg/l)	4.25 (1.7-13.8) ^### †††^	4.8 (2.7-10.9)	1.9 (0.5-6.0)	1.7 (0.5-7.1)

Comparison of the levels of plasma DPP4 activity and other clinical characteristics at diagnosis by classification based on disease type

Plasma DPP4 activity, reported as a potential predictor of DKD progression in new-onset T2DM [14], was compared at the time of SPIDDM diagnosis with that in AT1DM patients and healthy controls (Table 9). To ensure accurate comparisons, only individuals who had never used DPP4 inhibitors (DPP4i) during the study were included. In patients with SPIDDM (definite), plasma DPP4 activity was significantly higher than in those with AT1DM and healthy controls. It also showed a significant correlation with the UACR at the last outpatient clinic visit. Moreover, in this group, plasma DPP4 activity was significantly associated with BMI, CPI, hs-CRP levels at diagnosis, and the duration of DM prior to SPIDDM diagnosis. In SPIDDM (probable) patients and healthy controls, plasma DPP4 activity significantly correlated with BMI, and in healthy controls, it was also significantly associated with hs-CRP levels. These results suggest that plasma DPP4 activity is already high at the time of SPIDDM (definite) diagnosis and that it is correlated with albuminuria and metabolic syndrome-related factors.

**Table 9:** Comparison of the levels of plasma DPP4 activity and other clinical characteristics at diagnosis by classification based on disease type. Data are expressed as the mean ± standard deviation, the median (range), or number (%), and are compared using the student’s t-test, the Mann-Whitney U test, or Fisher's exact test. *p < 0.05 vs. SPIDDM (probable). #p < 0.05, ##p < 0.01, ###p < 0.001 vs. AT1DM. ††p < 0.01, †††p < 0.001 vs. Healthy controls. Pearson correlation analysis is performed to analyze the correlation between the levels of plasma DPP4 activity and other clinical characteristics. For this correlation analysis, non-normally distributed variables are log-transformed. All eligible patients did not use DPP4 inhibitors. Items without a time statement are at SPIDDM diagnosis. R, R correlation coefficient; SPIDDM, slowly progressive type 1 insulin-dependent diabetes mellitus; DM, diabetes mellitus; BMI, body mass index; SBP, systolic blood pressure; HbA1c, hemoglobin A1c; UACR, urine albumin-to-creatinine ratio; eGFR, estimated glomerular filtration rate; HDL-C, high-density lipoprotein cholesterol; LDL-C, low-density lipoprotein cholesterol; TG, triglycerides; UA, uric acid; hs-CRP, high-sensitivity C-reactive protein; AT1DM, acute-onset type 1 diabetes mellitus; N/A, not available; DPP4, dipeptidyl peptidase 4

	SPIDDM (definite)			SPIDDM (probable)			AT1DM			Healthy controls		
		Plasma DPP4 activity (Pearson correlation)			Plasma DPP4 activity (Pearson correlation)			Plasma DPP4 activity (Pearson correlation)			Plasma DPP4 activity (Pearson correlation)	
		R	P		R	P		R	P		R	P
Subjects (n)	43	N/A	N/A	7	N/A	N/A	21	N/A	N/A	50	N/A	N/A
Females (n, %)	25 (58) ^*^	N/A	N/A	1 (14)	N/A	N/A	10 (48)	N/A	N/A	22 (44)	N/A	N/A
Age at the last outpatient clinic (years)	60.2±16.5 ^##^	0.029	0.852	59.9±11.2	-0.546	0.205	46.8±15.0	0.392	0.0791	N/A	N/A	N/A
Age at the time of collecting serum (years)	50.1±16.1 ^## †††^	0.094	0.548	48.4±10.8	-0.414	0.356	36.4±13.1	0.293	0.198	61.8±8.49	-0.0767	0.597
BMI (kg/m^2^)	24.7±3.45 ^### ††^	0.603	<0.001	27.4±2.57	0.775	0.0407	21.1±2.77	0.29	0.202	22.5±2.89	0.377	0.007
SBP (mmHg)	122 (101-165) ^## ††^	0.121	0.440	131 (120-154)	-0.0335	0.943	113 (100-132)	0.0381	0.87	117 (94-138)	0.147	0.307
Duration of SPIDDM or AT1DM (years)	9 (1-24)	-0.094	0.548	12 (6-15)	-0.552	0.199	9 (2-34)	0.146	0.529	N/A	N/A	N/A
Duration of DM prior to the time of collecting serum (years)	4 (0-13) ^###^	0.616	<0.001	5 (0-12)	0.298	0.078	0 (0-8)	0.121	0.342	N/A	N/A	N/A
C-peptide index	1.13 (0.42-2.5) ^###^	-0.444	0.003	1.35 (0.84-2.17)	0.327	0.474	0.20 (0.07-0.5)	-0.034	0.884	N/A	N/A	N/A
HbA1c (%)	9.3 (6.5-14.1) ^## †††^	0.188	0.226	9.9 (7.5-13.2)	0.136	0.771	11.5 (7.1-16.6)	-0.301	0.185	5.6 (5.0-6.3)	0.0291	0.841
UACR at the time of collecting serum (mg/g)	4.5 (1.2-27)	-0.035	0.824	14.2 (2.2-32.9)	0.487	0.268	7.2 (1.1-69)	0.541	0.0113	N/A	N/A	N/A
eGFR at the time of collecting serum (ml/min/1.73m^2^)	76.4±12.6 ^### †^	-0.173	0.267	75.4±6.92	-0.291	0.526	103±32.5	-0.181	0.433	71.4±9.66	-0.15	0.298
UACR at the last outpatient clinic (mg/g)	38.8 (3.0-3000) ^###^	0.686	<0.001	43.8 (5.5-81.1)	0.836	0.0192	6.5 (1.9-1674)	0.31	0.171	N/A	N/A	N/A
eGFR at the last outpatient clinic (ml/min/1.73m^2^)	61.3±22.1 ^###^	-0.283	0.066	60.0±9.50	0.399	0.375	81.9±21.4	-0.292	0.198	N/A	N/A	N/A
hsCRP (mg/l)	4.6 (1.7-12.5) ^### †††^	0.439	0.003	6.3 (3.5-10.9)	0.608	0.147	1.9 (0.5-6.0)	0.11	0.636	1.7 (0.5-7.1)	0.283	0.0461
Plasma DPP4 activity (nmol/min/ml)	12.3±3.76 ^### †††^	N/A	N/A	10.1±1.32	N/A	N/A	9.22±2.28	N/A	N/A	7.13±2.09	N/A	N/A

Survival analysis of albuminuria in SPIDDM (definite) patients without DPP4 inhibitors

**Figure 6:**
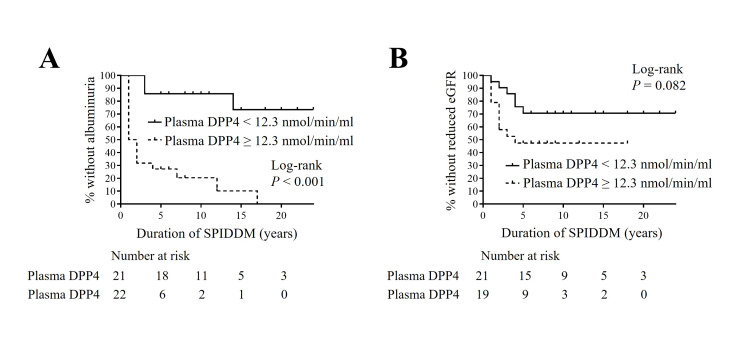
Kaplan-Meier survival curves for the new appearance of albuminuria (A) or the new decline in eGFR to less than 60 ml/minute/1.73 m2 (B) during follow-up for SPIDDM according to plasma DPP4 activity with cut-off ≤ 12.3 nmol/minute/ml. Items without a time statement are at SPIDDM diagnosis. Plasma DPP4, plasma dipeptidyl peptidase-4 activity; SPIDDM, slowly progressive type 1 insulin-dependent diabetes mellitus; eGFR, estimated glomerular filtration rate

Given that plasma DPP4 activity at SPIDDM diagnosis was significantly correlated with UACR at the last outpatient clinic, we conducted a survival analysis during the follow-up of SPIDDM to assess if plasma DPP4 activity could serve as a marker for future DKD progression. This analysis included 43 patients with SPIDDM (definite) who had not used DPP4i during the study. Initially, using the Kaplan-Meier method, we evaluated the long-term impact of plasma DPP4 activity on DKD progression (Figure 6). The patients were categorized into two groups based on the mean value of plasma DPP4 activity. The group with higher plasma DPP4 activity developed microalbuminuria significantly earlier than the lower activity group (Figure 6). However, no significant differences were observed in the progression to reduced eGFR between the two groups (Figure 6). Subsequently, both univariate and multivariate Cox proportional hazard analyses were performed, with plasma DPP4 activity as the covariate and the new onset of DKD as the event in SPIDDM (definite) patients not treated with DPP4i (Table 10). Plasma DPP4 activity demonstrated significant hazard ratios for the new appearance of albuminuria, even after adjustments for each parameter previously reported as a risk factor for DKD. These findings suggest that plasma DPP4 activity may be an influential factor associated with the new onset of albuminuria in SPIDDM.

**Table 10:** Cox proportional hazard models with plasma DPP4 activity as the covariate and with new-onset of DKD as the event in SPIDDM (definite) patients without DPP4 inhibitors. Items without a time statement are at SPIDDM diagnosis. SPIDDM, slowly progressive type 1 insulin-dependent diabetes mellitus; DM, diabetes mellitus; BMI, body mass index; SBP, systolic blood pressure; HbA1c, hemoglobin A1c; UACR, urine albumin-to-creatinine ratio; eGFR, estimated glomerular filtration rate; HDL-C, high-density lipoprotein cholesterol; LDL-C, low-density lipoprotein cholesterol; TG, triglycerides; UA, uric acid; hs-CRP, high-sensitivity C-reactive protein; DPP4, dipeptidyl peptidase 4; HR, hazard ratio; DKD, diabetic kidney disease

	Event: New appearance of albuminuria			Event: New decline in eGFR to less than 60 ml/minute/1.73 m^2^		
	Plasma DPP4 activity (nmol/minute/ml)			Plasma DPP4 activity (nmol/minute/ml)		
	HR	95% CI	P	HR	95% CI	P
Crude HR	1.312	1.151-1.495	< 0.001	1.093	0.946-1.264	0.228
Age-adjusted HR	1.310	1.149-1.494	< 0.001	1.118	0.962-1.298	0.145
Sex-adjusted HR	1.339	1.141-1.571	< 0.001	1.099	0.939-1.286	0.241
Smoking-adjusted HR	1.316	1.154-1.501	< 0.001	1.136	0.966-1.335	0.122
BMI-adjusted HR	1.200	1.044-1.380	0.010	1.047	0.891-1.230	0.574
SBP-adjusted HR	1.296	1.136-1.480	< 0.001	1.173	0.998-1.372	0.053
Duration of DM prior to SPIDDM diagnosis-adjusted HR	1.175	1.019-1.355	0.026	1.006	0.857-1.181	0.943
C-peptide index-adjusted HR	1.254	1.089-1.443	0.002	1.050	0.898-1.227	0.543
HbA1c-adjusted HR	1.311	1.149-1.496	< 0.001	1.125	0.968-1.307	0.125
UACR at SPIDDM diagnosis-adjusted HR	1.316	1.152-1.503	< 0.001	1.093	0.946-1.264	0.228
eGFR at SPIDDM diagnosis-adjusted HR	1.305	1.141-1.491	< 0.001	1.095	0.947-1.266	0.221
HDL-C-adjusted HR	1.317	1.151-1.508	< 0.001	1.088	0.943-1.256	0.248
LDL-C-adjusted HR	1.315	1.151-1.502	< 0.001	1.083	0.934-1.257	0.289
TG-adjusted HR	1.310	1.150-1.494	< 0.001	1.093	0.945-1.265	0.232
hs-CRP-adjusted HR	1.282	1.111-1.479	< 0.001	1.082	0.920-1.271	0.342

Survival analysis of albuminuria with and without the use of DPP4 inhibitors in patients with SPIDDM (definite)

**Figure 7:**
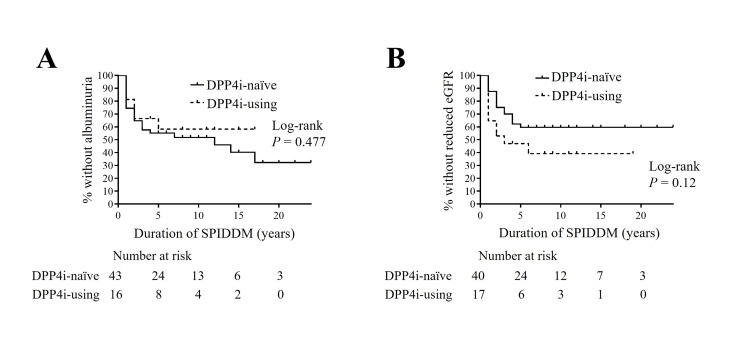
Kaplan-Meier survival curves for the new appearance of albuminuria (A) or the new decline in eGFR to less than 60 ml/minute/1.73 m2 (B) during follow-up for SPIDDM based on the use or non-use of DPP4 inhibitors. Items without a time statement are at SPIDDM diagnosis. DPP4i, dipeptidyl peptidase-4 inhibitor; SPIDDM, slowly progressive type 1 insulin-dependent diabetes mellitus; eGFR, estimated glomerular filtration rate

We further explored whether the use or non-use of DPP4i influenced DKD progression in SPIDDM. Initially, patients with SPIDDM (definite) were categorized into two groups: DPP4i-naïve (43 patients) and those using DPP4i (17 patients). The characteristics of these groups were then compared (Table 11). Consistent with previous findings, plasma DPP4 activity was significantly lower in DPP4i users compared to non-users. No significant differences in DKD diagnostic markers at the last outpatient clinic were observed between the two groups. Subsequently, we applied the Kaplan-Meier method to analyze these groups (Figure 7). There was no significant difference in the progression rate to albuminuria or reduced eGFR between the two groups. These results suggest that DPP4 inhibitors may not prevent DKD progression in patients with SPIDDM.

**Table 11:** Comparison of clinical characteristics with and without the use of DPP4 inhibitors in SPIDDM (definite) patients. Data are expressed as the mean ± standard deviation, the median (range), or number (%), and are compared using the student’s t-test, the Mann-Whitney U test, or Fisher's exact test. Items without a time statement are at SPIDDM diagnosis. DPP4i, DPP4 inhibitors; SPIDDM, slowly progressive type 1 insulin-dependent diabetes mellitus; DM, diabetes mellitus; BMI, body mass index; SBP, systolic blood pressure; HbA1c, hemoglobin A1c; UACR, urine albumin-to-creatinine ratio; eGFR, estimated glomerular filtration rate; HDL-C, high-density lipoprotein cholesterol; LDL-C, low-density lipoprotein cholesterol; TG, triglycerides; UA, uric acid; hs-CRP, high-sensitivity C-reactive protein; DPP4, dipeptidyl peptidase 4

	Overall (n=60)		
	DPP4i not used	DPP4i used	P
Subjects (n)	43	17	
Females, (n, %)	25 (58.1)	9 (52.9)	0.777
Age at the last outpatient clinic (years)	60.2±16.5	51.9±10.4	0.06
Age at SPIDDM diagnosis (years)	50.1±16.1	42.5±12.5	0.083
BMI (kg/m^2^)	24.7±3.45	24.1±4.40	0.554
SBP (mmHg)	122 (101-165)	143 (103-178)	0.475
Duration of SPIDDM (years)	9 (1-24)	8 (1-19)	0.73
Duration of DM prior to SPIDDM diagnosis (years)	4 (0-13)	5 (0-12)	0.558
C-peptide index	1.13 (0.42-2.5)	1.07 (0.61-2.9)	0.883
HbA1c (%)	9.3 (6.5-14.1)	9.3 (7.3-15.9)	0.799
UACR at SPIDDM diagnosis (mg/g)	4.5 (1.2-27)	5.7 (1.0-211)	0.85
eGFR at SPIDDM diagnosis (ml/min/1.73m^2^)	76.4±12.6	81.8±11.6	0.136
UACR at the last outpatient clinic (mg/g)	38.8 (3.0-3000)	19.6 (3.3-2000)	0.533
eGFR at the last outpatient clinic (ml/min/1.73m^2^)	61.3±22.1	53.6±21.3	0.224
hs-CRP (mg/l)	4.6 (1.7-12.5)	4.1 (1.7-13.8)	0.367
Plasma DPP4 activity (nmol/min/ml)	12.3±3.76	6.37±2.93	<0.001

## Discussion

This study explored whether characteristics and laboratory findings at the time of SPIDDM diagnosis could be linked to the progression of DKD. We found that BMI and hs-CRP levels at SPIDDM diagnosis, along with the duration of DM prior to SPIDDM diagnosis, were associated with the new onset of albuminuria. In contrast, SBP at SPIDDM diagnosis and the duration of DM prior to SPIDDM diagnosis were associated with a new decline in eGFR to less than 60 ml/minute/1.73 m². Furthermore, the patients with SPIDDM were found to already have a high inflammatory state at the time of diagnosis. Finally, plasma DPP4 activity was linked to the new appearance of albuminuria in patients with SPIDDM who were not treated with DPP4i. This study is novel in its revelation of aspects of the pathogenesis of DKD progression in SPIDDM.

Interestingly, while the duration of SPIDDM itself was not associated with DKD progression, the duration of DM prior to SPIDDM diagnosis was a significant factor. This aligns with previous findings suggesting that the duration of DM is a predictor of DKD progression in T2DM patients [7,21]. Furthermore, prolonged DM duration has been shown to promote chronic inflammation, exacerbating diabetic complications [22]. The significant positive correlation between the duration of DM prior to SPIDDM diagnosis and hs-CRP levels underscores this relationship. These findings highlight the importance of early diagnosis and treatment of SPIDDM for renal protection.

In this study, BMI and SBP at the time of SPIDDM diagnosis were identified as factors associated with DKD progression in SPIDDM. Notably, both BMI and SBP have been previously recognized as predictors of DKD in T2DM [23,24]. Additionally, hs-CRP levels at SPIDDM diagnosis were also associated with DKD progression. hs-CRP has been documented to predict DKD progression in patients with AT1DM and T2DM [11, 12]. Patients with SPIDDM are often already being treated as DM patients for a long period of time at the time of diagnosis. In this respect, they differ from AT1DM in their characteristics. This feature in SPIDDM may contribute to the already high inflammatory status at the time of SPIDDM diagnosis. Given that microinflammation is a known progression factor in DN [25], a high inflammatory state may similarly predict subsequent DKD progression in SPIDDM patients.

This study further provides evidence that plasma DPP4 activity at SPIDDM diagnosis is linked to DKD progression. Blood soluble DPP4 (sDPP4) levels and plasma DPP4 activity have been identified as potential predictors of DKD progression in new-onset T2DM [14,26]. Moreover, a recent study in mice in which DN was induced in an inflammatory manner by genetically engineered overexpression of the inflammatory marker CRP reported that DPP4 was involved in the pathogenesis of DN associated with exacerbated inflammation [27]. In our findings, plasma DPP4 activity was associated with the new appearance of albuminuria. These data suggest that DPP4 may contribute to DKD progression, raising a clinical question regarding whether DPP4i could help suppress the progression of DN in patients with SPIDDM.

The outcomes of previous large clinical trials have left the renoprotective effects of DPP4i somewhat ambiguous. A secondary analysis of the Saxagliptin Assessment of Vascular Outcomes Recorded in Patients with Diabetes Mellitus (SAVOR)-Thrombolysis in Myocardial Infarction (TIMI) 53 (SAVOR-TIMI53) trial, which examined saxagliptin, noted a reduction in albuminuria over a two-year period but found no improvements in eGFR within the same group [28]. Similarly, the Efficacy, Safety & Modification of Albuminuria in Type 2 Diabetes Subjects with Renal Disease with LINAgliptin (MARLINA-T2D) trial with linagliptin did not demonstrate any renoprotective effects, such as changes in UACR or eGFR [29]. In our study, no significant differences were found in DKD progression markers among SPIDDM patients treated with or without DPP4i. These findings suggest that while DPP4 may be a factor associated with DKD progression in SPIDDM, the extent of DPP4 suppression by DPP4i does not seem to offer renal protection. Moreover, the retrospective nature of this study means that the possibility of selection bias, where DPP4i was potentially prescribed more frequently to patients at higher risk of DKD progression, cannot be dismissed.

In our analysis, plasma DPP4 activity was significantly elevated in patients with SPIDDM (definite) compared to those with AT1DM and healthy controls. DPP4, an adipokine secreted by adipocytes, is induced by inflammation and obesity [27,30]. Patients with SPIDDM exhibited a longer duration of DM prior to diagnosis, higher levels of inflammation, and increased BMI, which could explain the heightened plasma DPP4 activity observed.

This study faces two main limitations. Firstly, due to its retrospective design, there was an inherent treatment selection bias, which left the initiation of insulin and renoprotective agents to individual physicians. This might explain the lack of significant differences in prescribed medications for SPIDDM patients based on the severity of DKD, despite evidence of certain medications like sodium-glucose cotransporter 2 inhibitors, glucagon-like peptide-1 receptor agonists, angiotensin-converting enzyme inhibitors, and angiotensin receptor blockers suppressing DKD progression in T2DM patients. Secondly, the small number of patients with SPIDDM in this study prevented the simultaneous entry of all items known as risk factors for kidney disease, such as age, gender, smoking habits, and dyslipidemia, into the multivariate analysis, potentially weakening the statistical power of the findings. Future studies, ideally with larger cohorts and a prospective design at the time of diagnosis, are needed.

Our findings highlight the importance of early diagnosis and management of SPIDDM to prevent DKD progression. The novel insights from this study could be crucial in understanding the pathogenesis of DKD in SPIDDM patients.

## Conclusions

In conclusion, this study identified BMI, SBP, hs-CRP, DPP4 activity at the time of SPIDDM diagnosis, and duration of DM prior to SPIDDM diagnosis as factors associated with the appearance of DKD in patients with SPIDDM. Also, we found that the patients with SPIDDM are often already being treated as DM patients for a long period of time at the time of diagnosis, which may be linked to their already high inflammatory status at the time of SPIDDM diagnosis and contribute to DKD progression. Recognizing these risk factors early on may guide the clinical approach toward renal protection from the outset of SPIDDM diagnosis.
